# When the Underdog Apologizes: The Role of Intergroup Apologies in Historical Intergroup Conflicts

**DOI:** 10.5334/irsp.1157

**Published:** 2026-04-08

**Authors:** Alin Semenescu

**Affiliations:** 1 West University of Timisoara, Romania

**Keywords:** intergroup apologies, intergroup contact, intergroup conflict, reconciliation, reversed apology

## Abstract

Addressing ongoing intergroup conflicts remains a major challenge for social psychologists. The present vignette-based experiment (N = 178) examined a historical conflict between Romanians (the majority group) and Romas (the historically disadvantaged group), exploring the effects of intergroup apologies issued by the disadvantaged group, intergroup contact, and their interaction on forgiveness and trust toward the Romas. The perceived sincerity of the apology and the perceived typicality of the apologizing representatives were examined as potential factors shaping the relationship between the apology and these outcomes. Results show that intergroup contact was positively associated with both forgiveness and trust, while apologies led to increased trust. Perceiving the apology as sincere was associated with greater forgiveness, whereas perceiving the apologizing representatives as typical of their group predicted higher levels of both forgiveness and trust, highlighting the central role of recipients’ perceptions. Present findings emphasize that low-power groups can actively contribute to reconciliation processes and provide practical insights for practitioners working in conflict-affected societies.

## 1. Introduction

The 20^th^ century, the bloodiest in human history, saw the deaths of over 231 million people in armed group conflicts (Leitenberg 2006). As disturbing as this number may be, group conflicts are far from a relic of the past; they continue to persist—and even escalate—in numerous contemporary societies, with the recent war in Ukraine standing out as a striking example. Most often they take milder forms of violence such as prejudice or discrimination towards certain groups (e.g., the growing discrimination towards Muslims in Western Europe), though they can at times escalate into more extreme expressions, such as attempts to physically exterminate one group by another (e.g., the genocide in Rwanda when two-thirds of Tutsis were murdered by Hutus).

Compared to interpersonal conflicts, intergroup conflicts involve large numbers of people, are often more severe, last longer (sometimes for decades or centuries), are ideologically supported, and involve a high degree of mistrust (Wohl et al. 2011). Among the practical actions that socio-psychological research suggests would be effective for ameliorating intergroup relations are *intergroup apologies* and *intergroup contact*. Although their positive outcomes have largely been observed in relatively mild intergroup contexts, the encouraging results offer hope that these strategies could also be effective in more intense or severe conflict situations (see, for example, Hewstone et al. 2014; Voci et al. 2015). The present study was conducted in Romania, a context characterized by a long-standing and deeply entrenched intergroup conflict between ethnic Romanians (the majority) and the Roma community (a minority group), a tension that has endured for centuries. Although the harm experienced by the two groups is clearly asymmetrical, with Roma subjected to centuries of structural and institutionalized oppression (e.g., five centuries of slavery and their persecution during World War II), whereas Romanians have more commonly experienced interpersonal and symbolic harms (e.g., threats to their collective identity because of being associated with the Roma), both groups tend to view themselves as victims, a pattern that is common in protracted historical conflicts. Typically, the main perpetrator is the one expected to apologize. Although ethnic Romanians have historically and contemporarily been the primary perpetrators of violence in this relationship, the present research examined Romanian participants’ responses to an unexpected apology issued by the Roma group. Using a vignette-based experimental design, the study investigated the effects of *intergroup apologies* (experimentally manipulated) and *intergroup contact* (measured) on Romanians’ *forgiveness* and *trust* toward the Roma.

### 1.1. Intergroup apologies

Often, the first contemplated action is revenge when an individual or group is harmed by another (Rusbult et al. 1991). While seeking revenge may help the victim regain a sense of control and self-worth (Akhtar 2002), it often comes at a substantial cost to the relationship. Among others, it may serve as a pretext for subsequent retaliation, leading to a downward spiral of aggression and a progressive erosion of the relationship. One way to prevent retaliation following an unjust harm is through offering an apology. Intergroup apologies are public acknowledgments made by representatives of a group (Tavuchis 1991), indicating that the offending group recognizes its role in the conflict and is committed to addressing past wrongs. For the victim group, such apologies are important because they publicly affirm the injustice suffered and serve as a record by which the offending group can be held accountable for its future actions.

Some scholars view intergroup apologies as pivotal moments in the relationship between conflicting groups, as they can represent a meaningful turning point and the beginning of a renewed interaction (Wohl et al. 2011). Some of the most famous examples include the Canadian government’s apology to Aboriginal Canadians for the abusive residential school system or the Australian Prime Minister’s apology to the ‘Stolen Generations’ of Aboriginal people who were removed as children from their families (Wohl et al. 2011). In fact, apologies have been used so often in the past decades by governments, group leaders or corporations that some authors have begun speaking about an ‘age of apology’ (e.g., Brooks 1999).

It was extensively argued by theorists that intergroup apologies can be beneficial for intergroup relations (e.g., Lazare 2005; Tavuchis 1991), which led to a widely held presumption that intergroup apologies represent a key element for reconciliation (Wohl et al. 2011). The recipients of apologies themselves seem to share such views: For example, 93% of Aboriginal Australians rated the Australian government’s apology as very important for its abuses against the Aboriginal community, and 80% rated it as necessary for promoting amiable relations with Australians (Wohl et al. 2011). Apologies’ positive effects have been observed on outcomes such as improved cooperation with the offender (Halabi et al. 2013), decreased desire for retribution (Leonard et al. 2011), greater willingness for contact (Borinca et al. 2024) or more positive evaluations of the perpetrator (Blatz et al. 2014). However, when studied against more stringent criteria such as forgiveness or trust, the few existing experimental studies revealed conflicting results. For example, in Study 1, Brown et al. (2008) found that a U.S. apology for a friendly fire incident in Afghanistan, which resulted in the deaths of Canadian soldiers, led to increased forgiveness of the U.S. military among Canadian participants. Similarly, Leonard et al. (2011) found that an apology encouraged students to forgive an insult made by faculty members, while Reinders Folmer et al. (2021) showed that following a transgression in which a group of students kept most of the money in a Dictator game, offering an apology increased the victim group’s trust in the transgressors. A similar result was found by Ma et al. (2019), who showed that participants receiving an apology were more likely to trust their transgressors after a trust violation. However, other experimental studies have not supported these findings. In four experiments, Philpot and Hornsey (2008) observed that while an apology enhanced perceptions of remorse and increased victims’ satisfaction, it did not lead to greater forgiveness. Similarly, Steele and Blatz (2014), in five different apology conditions, found no effect of apologies on forgiveness or trust, while Kachanoff et al. (2017) found no significant effect of apologies on forgiveness.

One of the most widely used frameworks for understanding when apologies are effective is the needs-based model of reconciliation (Shnabel & Nadler 2008). This model proposes that reconciliation depends on the reciprocal fulfillment of the emotional needs of both victims and perpetrators. Specifically, victims are thought to experience a threat to their sense of power, whereas perpetrators are thought to experience a threat to their moral identity. Consequently, reconciliation is facilitated when perpetrators help restore victims’ sense of power and when victims affirm the perpetrators’ moral image.

While the needs-based model emphasizes emotional needs rooted in the roles of victim and perpetrator, other scholars have highlighted the importance of cognitive factors in shaping the effectiveness of apologies. For example, Wohl et al. (2013) argued that the perceived *sincerity* of the apology is crucial for its acceptance by the victim. If the perpetrator group is viewed as insincere, their apology may be perceived as a cynical tactic rather than a genuine expression of remorse (Wenzel et al. 2017). A sincere apology may also humanize the apologizing group by signaling to the victimized group that its members are capable of experiencing uniquely human emotions, such as remorse (Borinca et al. 2021; Wohl et al. 2012), thereby eliciting emotional responses, such as reduced anger or increased respect, that are conducive to positive intergroup outcomes (Leonard et al. 2011). Supporting this, Wohl et al. (2013) found that perceptions of sincerity in an official apology significantly predicted forgiveness among Chinese Canadians toward European Canadians for the head tax imposed on Chinese immigrants. Similarly, Wenzel et al. (2017) showed that higher perceptions of apology sincerity increased forgiveness toward the transgressor group, while Borinca et al. (2021) found that apologies perceived as genuine were successful in promoting positive intergroup outcomes.

Another important cognitive aspect concerns perceptions of the *typicality* of the apologizing representatives. Since the perpetrator group as a whole shares responsibility for the transgression (Wohl et al. 2006), those delivering the apology must be seen as legitimately representing the group on whose behalf they speak. Group members are generally conscious of the variability existing in outgroups, even though it is to a lesser extent than in their own (Quattrone & Jones 1980). Out-groups often consist of subgroups that vary across multiple dimensions, and these subgroups may also differ in their stance toward supporting an apology, which can significantly influence the apology’s effectiveness. For instance, perceiving the out-group as a coherent and unified entity (i.e., high entitativity) (Campbell 1958) resulted in viewing its apology as more sincere and less driven by ulterior motives, compared to apologies issued by out-groups perceived as low in entitativity (Leonard 2019). Similarly, Wenzel et al. (2017) demonstrated that apologies perceived as highly representative of the offending group elicited greater forgiveness than those perceived as less representative. In their study, victims were informed about the degree of support for the apology within the offender group, such as the proportion of group members endorsing it. In real-world contexts, however, victims rarely have access to such information and typically rely solely on the message conveyed by the apologizing representatives. Consequently, it is essential that these representatives are perceived as genuine members of and authorized spokespeople for the transgressor group. Even subtle discrepancies between the apologizers and the prototypical image of the offending group can activate subtyping processes (Taylor 2015), leading victims to interpret the apology as originating from an exceptional subgroup rather than from the group as a whole. In other words, apologizing representatives need to be perceived as typical members of the offender group. Although the mechanisms of perceived sincerity and typicality have traditionally been examined in contexts where apologies come from more powerful to less powerful groups, they are likely to operate similarly in reversed power dynamics, such as those investigated in the present study.

One of the objectives of the present study is to examine how an intergroup apology affects forgiveness and trust toward the apologizing group and whether this effect is influenced by the perceived sincerity of the apology and the perceived typicality of the apologizing representatives within the context of a real-world conflict between Romas (the apologizing group) and Romanians (the recipient group). A distinctive feature of this research lies in its reversed direction of apology—from the marginalized to the powerful group—allowing an investigation into whether members of less powerful victim groups can take an active role in reconciliation. Nadler and Liviatan (2006) investigated a similar dynamic in the context of a non-apologetic speech and found that expressions of empathy by a Palestinian authority (the lower-power group) increased Israelis’ (the higher-power group) willingness to reconcile but only among those who reported high levels of trust in Palestinians. To the best of my knowledge, however, the present study is the first empirical study to directly examine the effects of apologies issued by the less powerful group. These dynamics have been observed in real-world contexts as well. For example, following the end of apartheid, leaders of the African National Congress issued apologies to white South Africans—the former oppressors—for violent acts committed during the anti-apartheid struggle (Institute for the Study of Human Rights n.d.). Similarly, in the context of the Israeli–Palestinian conflict, Palestinian authorities have at various points apologized for attacks targeting Israeli civilians (e.g., Yang & Sagalyn 2023). However, despite these real-world examples, the extent to which such apologies are actually effective remains a question that needs to be empirically investigated.

### 1.2. Intergroup contact

Aside from intergroup apologies, intergroup contact was often used to improve intergroup relations. Allport (1954) was one of the first social psychologists to suggest that intergroup contact is conducive to improved intergroup outcomes. He specified four conditions under which contact leads to improved intergroup attitudes, namely, equal status, authority support, working towards a common goal, and cooperation towards reaching that goal. Research has generally supported Allport’s claims, although a later meta-analysis by Pettigrew and Tropp (2006) has provided evidence that intergroup contact is associated with more favorable intergroup attitudes irrespective if these conditions are met or not.

The positive effects of intergroup contact were observed for a number of intergroup outcomes, such as prejudicial attitudes (Hamberger & Hewstone 1997), anger reduction (Tam et al. 2007), as well as trust (Vezzali et al. 2012; Tausch et al. 2011) and forgiveness (Tam et al. 2007) and for diverse targets, such as ethnic minorities (Hamberger & Hewstone 1997), homosexuals (Herek & Capitanio 1996), or people with HIV/AIDS (Werth & Lord 1992), to name a few. This compelling evidence supports the use of intergroup contact as a primary intervention strategy when addressing problematic intergroup relations. However, caution is necessary when such contact occurs without meeting Allport’s optimal conditions, as research indicates that negative intergroup interactions can actually worsen intergroup attitudes (Techakesari et al. 2015).

As Hewstone et al. (2014) point out, with some exceptions (for a review, see Al Ramiah and Hewstone 2013), the positive effects of intergroup contact have largely been examined in relatively benign intergroup contexts. However, the situation may be quite different in contexts marked by historical conflict, where the conflict itself and negative perceptions of the opposing group have become embedded in the group’s collective ideology (Bar-Tal et al. 2012). Such ideologies can shape how individuals interpret intergroup interactions, potentially leading to more negative appraisals during and after contact and even reinforcing hostile attitudes toward the outgroup (Hayward et al. 2017). Nonetheless, several studies offer encouraging findings from conflictual contexts. In Northern Ireland, Tam et al. (2009) found that intergroup contact predicted more positive behavioral intentions toward the outgroup, while Tausch et al. (2011) showed that extended contact fostered outgroup trust. In Israel, Maoz and Ellis (2008) reported that Israeli Jews who engaged in structured dialogue with Palestinians expressed greater trust and stronger support for compromise-based solutions to the Israeli-Palestinian conflict. Similarly, in Bosnia and Herzegovina, Cehajic et al. (2008) found that intergroup contact was positively associated with Bosnian Muslims’ willingness to forgive Bosnian Serbs for wartime transgressions. Together, this compelling evidence suggests that, when the right conditions are met, intergroup contact can promote improved intergroup relations even in deeply divided contexts. To strengthen the validity of these findings, it is essential to further examine the positive effects of contact in real-world settings characterized by serious and ongoing conflict, such as the one addressed in the present study.

### 1.3. Interaction between apology and contact

Notably, the combined effects of intergroup contact and intergroup apologies have, to the best of my knowledge, not yet been examined. Beyond acknowledging wrongdoing, an apology may also signal a desire to restore damaged relations (Hareli & Eisikovits 2006). In a collectivistic context strongly focused on relationships such as the Romanian one (Gavreliuc 2011), living harmoniously with one’s neighbors and acquaintances is part of the cultural mandate and is highly valued. As such, Romanians who have frequent contact with Romas may have an added motivation to restore relations, which could further encourage acceptance of the Roma group’s apology.

Second, intergroup contact has been shown to impact perceptions of common humanity (Capozza et al. 2014), thus creating the psychological conditions under which an apology can be seen as a credible gesture from a humane group. Additionally, contact can enhance cognitive differentiation and perspective-taking, which are processes associated with integrative complexity, defined as the ability to differentiate between multiple viewpoints and to integrate them into a coherent whole (Tetlock 1985). Consequently, high-contact individuals should show less black-and-white moral reasoning regarding Roma behavior and rely less on the default distrust and suspicion that characterizes this intergroup context. Instead, they may be more inclined to attribute the apology to genuine remorse, rather than a strategic move from an adversarial group. Thus, intergroup contact and apology may interact, with the apology potentially having a stronger positive effect on those Romanians who have greater contact with Romas compared to those with limited contact.

### 1.4. The present study

In long-standing intergroup conflicts, both parties often assume the roles of both victims and perpetrators of violence. The present study focuses on the perspective of Romanians and examines the effects of apologies issued by the historically marginalized group (i.e., Romas), the effect of intergroup contact, and their interaction on forgiveness and trust toward the Romas. Additionally, it explores whether the perceived sincerity of the apology and the perceived typicality of the apologizing representatives influence the relationship between the apology and the outcome variables (forgiveness and trust). Drawing on the reviewed literature, the following hypotheses are proposed:

*H1: Intergroup apologies will lead to increased forgiveness toward the less powerful group*.

*H2: Perceived sincerity of the apology will positively predict forgiveness toward the less powerful group*.

*H3: Perceived typicality of the apologizing representatives will positively predict forgiveness toward the less powerful group*.

*H4: Intergroup apologies will lead to increased trust in the less powerful group*.

*H5: Perceived sincerity of the apology will positively predict trust in the less powerful group*.

*H6: Perceived typicality of the apologizing representatives will positively predict trust in the less powerful group*.

*H7: Intergroup contact will be positively associated with forgiveness of the less powerful group*.

*H8: Intergroup contact will be positively associated with trust in the less powerful group*.

*H9: Apology’s effect on forgiveness will be moderated by intergroup contact*.

*H10: Apology’s effect on trust will be moderated by intergroup contact*.

## 2. Method

### 2.1. Ethical statement

The study was conducted in accordance with the ethical principles outlined in the Declaration of Helsinki and was approved by the Ethics Committee of the West University of Timișoara. When requesting approval, a short description of the study, together with the method of data collection and expected results were provided.

### 2.2. Power analysis

Sample size was calibrated using G*Power, version 3.1.9.4 (Faul et al. 2007). To detect a small-to-medium effect size of *d* = 0.40, for α = 0.05 and a power of 0.80, the required sample size was N = 156. This value acted as a benchmark against which the study was calibrated.

### 2.3. Design

The study employed a 2 (contact) × 2 (apology) between-subjects quasi-experimental design, with contact treated as a measured variable and apology as a manipulated variable.

### 2.4. Participants and procedure

The study was conducted in the city of Timisoara (approximately 320,000 inhabitants), in western Romania. Participants were approached by the same person in parks, bars, festivals, churches, public universities, or the city center and asked to complete one of the two versions of a paper-and-pencil questionnaire. Such a variety of places was used so that the resulting sample is fairly representative of the general population of the city. All participants gave their informed consent for participation and were debriefed at the end of the study. Of the 339 persons approached, 196 agreed to complete the questionnaire, resulting in a participation rate of 57.8%. Of these, 18 participants were removed from further analyses on the following considerations: Twelve were not of Romanian ethnicity, two failed to follow the instructions, and four misidentified the article’s topic used in the manipulation. Therefore, the final sample consisted of 178 Romanian ethnics, which ranged in age from 16 to 67 years (*M* = 31.85, *SD* = 11.32). In terms of income, 13.5% declared below minimum income, 38.2% below average, 43.8% above average, and 4.5% did not state their income. Regarding education, 0.6% stated primary school, 1.7% secondary school, 24.2% high school, 6.2% post-high school studies, 42.1% bachelor, and 24.2% postgraduate studies as their highest educational achievement, while 1.1% did not declare their highest educational achievement.

### 2.5. Measures

*Apology* was manipulated using a printed online article embedded within the questionnaire, presented before the dependent variables were measured, and randomly assigned to approximately half of the participants (N = 87) (see Image 1 in Supporting Information). The article appeared to originate from a well-known Romanian news agency and was written in a style consistent with that of the publication. The apology was conceived to contain an expression of remorse (Nunney & Manstead 2021), an implied admission of responsibility (see Tavuchis 1991), as well as a stated desire to restore and strengthen the relationship with the victim group (see Hareli & Eisikovits 2006). Additionally, given that research suggests that costly apologies are perceived as more effective than less costly ones (Blatz & Philpot 2010), the article included a statement highlighting the symbolic costs for the Romas (see Supporting Information for the full text of the apology). Three items were used to determine if the apology manipulation was successful, each measured on a 5-point scale (1 = strongly disagree, 5 = strongly agree): ‘I think that Romas are sorry for their wrongdoings’ (*remorse*), ‘I believe that Romas took some responsibility for their wrongdoings’, (*responsibility*) and ‘I believe that Romas intend to strengthen their relationship with Romanians’ (*restore relationship*).

*Contact* with the apologizing group was measured following the approach of Voci and Hewstone (2003), assessing both the quality (‘In general, when you meet Romas, do you find the contact rather positive or negative?’; 1 = very negative, 5 = very positive) and quantity (‘How much contact have you had with Romas at home, work, school, or in social situations?’; 1 = not at all, 5 = a great deal) of contact. In line with their approach, a composite score was created by multiplying quality by quantity of contact.

*Forgiveness* was measured with three items adapted from Wohl et al. (2012), such as ‘It is important that Romanians never forgive the wrongs done by the Romas’ (1 = strongly disagree, 5 = strongly agree; α = .79).

*Trust* was assessed with three items adapted from Noor et al. (2008), including ‘Few Romas can be trusted’ (1 = strongly disagree, 5 = strongly agree; α = .75).

Perceived *sincerity* of the apology and perceived *typicality* of the apologizing representatives were each measured with a single item (1 = strongly disagree, 5 = strongly agree): ‘I believe that Romas’ apologies that I read in the article were sincere’ (sincerity) and ‘I believe that the Romas that apologized in the article are representative of Romas in general’ (typicality).

## 3. Results

### 3.1. Manipulation checks

Success of randomization was checked by comparing the *apology* (N = 87) with the *no-apology* (N = 91) conditions on all demographics and on the contact variables. No significant differences between the two conditions emerged on all studied outcomes (all *p*s > 0.194), indicating that randomization was successful. Next, the *apology* and *no-apology* conditions were compared based on the three manipulation check measures. There were significant differences between the conditions regarding perceptions of remorse (*mean difference* = 0.47, *t*(176) = 3.21, *p* = 0.002, *d* = 0.48), perceptions of assumed responsibility by the Romas (*mean difference* = 0.46, *t*(176) = 2.90, *p* = 0.004, *d* = 0.43), and perceptions that the Romas intend to restore their relationship with Romanians (*mean difference* = 0.48, *t*(176) = 3.05, *p* = 0.003, *d* = 0.45). These results indicate that the manipulation was successful and produced effects of moderate magnitude.

### 3.2. Hypotheses testing

To test whether intergroup apologies lead to increased forgiveness (H1) and trust (H4) toward the less powerful group, two independent samples t-tests were conducted, in which the *apology* and *no-apology* conditions were compared on *forgiveness* and *trust*. Normality was examined for each group, and no substantial deviations from a normal distribution were detected. There was no significant difference between the apology (*M* = 3.85, *SD* = 0.76) and no-apology (*M* = 3.78, *SD* = 0.93) conditions regarding forgiveness, *t*(176) = 0.55, *p* = 0.291, *d* = 0.08, indicating that the apology had no impact on forgiveness of Romas. Therefore, H1 was not supported by the data. However, there was a significant difference between the apology (*M* = 2.85, *SD* = 0.77) and no-apology (*M* = 2.56, *SD* = 0.69) conditions regarding trust, *t*(176) = 2.62, *p* = 0.005, *d* = 0.40. This suggests that participants who received the apology expressed significantly higher trust in Romas, compared to those who did not, providing support for H4.

To examine whether perceived sincerity of the apology positively predicts forgiveness (H2) and trust (H5), and whether perceived typicality of the apologizing representatives positively predicts forgiveness (H3) and trust (H6)*, sincerity* and *typicality* were entered simultaneously into two different regression models predicting *forgiveness* and *trust*. These analyses were conducted exclusively on participants exposed to the apology manipulation (N = 87), as sincerity and typicality were not measured in the no-apology condition. Analyses of linearity, normality of residuals, and homoscedasticity revealed no serious violations. There were also no multicolinearity issues between predictors (VIF < 4). In the model predicting forgiveness, sincerity emerged as a significant predictor (*β* = 0.54, *p* < 0.001), supporting H2, whereas typicality was non-significant (*β* = 0.13, *p* = 0.090), failing to support H3. Together, the predictors accounted for approximately 37% of the variance in forgiveness (*R²* = 0.365). In the model predicting trust, both sincerity (*β* = 0.29, *p* = 0.003) and typicality (*β* = 0.35, *p* < 0.001) were significant predictors, thereby supporting H5 and H6. The model explained about 29% of the variance in trust (R² = 0.289) (see Table 1).

**Table 1:** Regression coefficients of sincerity and typicality when regressed on forgiveness and trust.


	β	*P*	*R^2^*

Forgiveness			0.365

Sincerity	0.54	<0.001	

Typicality	0.13	0.090	

Trust			0.289

Sincerity	0.29	0.003	

Typicality	0.35	<0.001	


**Figure 1:**
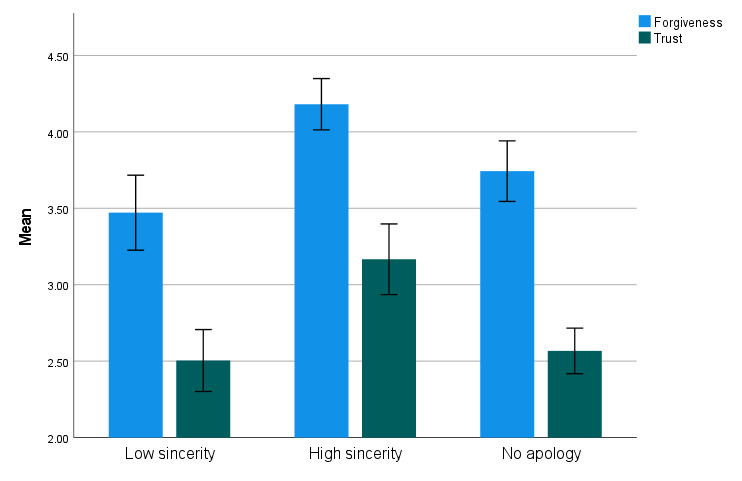
Forgiveness and trust in the perpetrator as a function of the perceived sincerity of the apology.

To further examine how apology effectiveness varied with perceived *sincerity* and *typicality*, the no apology, low-sincerity/typicality, and high-sincerity/typicality groups were compared. For this purpose, sincerity and typicality were first dichotomized based on their respective mean scores. Participants scoring below the mean were assigned to the low group, while those scoring above the mean were assigned to the high group (see Table 2 for group means and standard deviations). Assumptions checks revealed no substantial deviations from normality and no violations of homoscedasticity. Four ANOVAs were then conducted to evaluate differences between the low, high, and no-apology groups for both sincerity and typicality. Post-hoc tests with LSD correction were applied. Regarding the effect of *sincerity* on *forgiveness*, participants in the high-sincerity group reported significantly greater forgiveness than those in the no-apology group, *t*(172) = 2.96, *p* = 0.004, *d* = 0.56, and the low-sincerity group, *t*(172) = 4.08, *p* < 0.001, *d* = 1.04. Interestingly, an almost significant boomerang effect emerged for insincere apologies, as participants in the low-sincerity group displayed lower forgiveness levels than those in the no-apology condition, *t*(172) = –1.79, *p* = 0.075, *d* = –0.33 (see Figure 1). Regarding the effect of *sincerity* on *trust*, participants in the high-sincerity group reported significantly greater trust than those in the no-apology condition, *t*(173) = 4.70, *p* < 0.001, *d* = 0.81, and the low-sincerity group, *t*(173) = 4.39, *p* < 0.001, *d* = 0.94. No significant difference was found between the no-apology and low-sincerity groups regarding trust, *t*(173) = 0.46, *p* = 0.644, *d* = 0.11.

**Table 2:** Means and standard deviations of forgiveness and trust as a function of sincerity and typicality.


	APOLOGY (N = 87)								NO APOLOGY	
	
	HIGH SINCERITY (*N*= 46)		LOW SINCERITY (*N*= 41)		HIGH TYPICALITY (*N*= 50)		LOW TYPICALITY (*N*= 37)		(N = 91)	
	
	*M*	*SD*	*M*	*SD*	*M*	*SD*	*M*	*SD*	*M*	*SD*

Forgiveness	4.18	0.57	3.47	0.78	3.96	0.68	3.69	0.84	3.74	0.93

Trust	3.17	0.78	2.50	0.64	3.08	0.77	2.55	0.71	2.56	0.69


**Figure 2:**
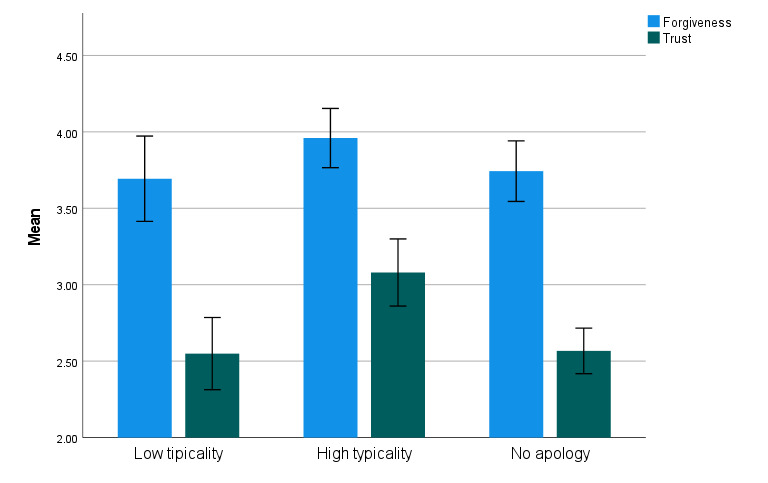
Forgiveness and trust in the perpetrator as a function of the typicality of the apologizing representatives.

Concerning the effect of *typicality* on *forgiveness*, there were no significant differences in forgiveness between the three groups (i.e., all *p*s > 0.05). However, significant differences in *trust* emerged between the three typicality groups: participants in the high-typicality group reported significantly greater trust than those in both the no-apology group, *t*(173) = 4.06, *p* < 0.001, *d* = 0.70, and the low-typicality group, *t*(173) = 3.40, *p* < 0.001, *d* = 0.72. No significant difference in trust was observed between the no-apology and low-typicality groups, *t*(173) = 0.11, *p* = 0.910, *d* = 0.01 (see Figure 2). These findings underscore the critical importance of recipients’ perceptions regarding the apology.

To test whether intergroup contact is positively associated with forgiveness (H7) and trust in the less powerful group (H8), *contact* was regressed on *forgiveness* and *trust*. Checks revealed no serious violations of model assumptions. Forgiveness was significantly predicted by contact (*β* = 0.24, *p* < 0.001), indicating that greater contact was associated with more forgiveness, thus supporting H7. Similarly, trust was significantly predicted by contact (*β* = 0.33, *p* < 0.001), indicating that greater contact was associated with higher trust, thereby supporting H8.

To examine whether intergroup contact moderates the relationship between apology and forgiveness (H9) and between apology and trust (H10), the interaction between *contact* and *apology* was investigated for both *forgiveness* and *trust*. For this purpose, two regression models were conducted in which apology, contact, and their interaction were introduced as predictors for forgiveness and trust, respectively. The interaction terms between contact and apology were non-significant for both forgiveness, *β* = 0.21, *t*(171) = 0.98, *p* = 0.329, and trust, *β* = 0.09, *t*(171) = 0.42, *p* = 0.678. Therefore, H9 and H10 were not supported by the data.

## 4. Discussion

The present study examined the effects of intergroup apologies from a historically marginalized group, those of intergroup contact, and their interaction on intergroup forgiveness and trust within the context of a real-world intergroup conflict. It also explored how the perceived sincerity of the apology and the perceived typicality of the apologizing representatives influenced its effectiveness.

Regarding intergroup contact, the results indicated that repeated, positive contact with Romas was associated with greater forgiveness and trust, aligning with extensive evidence that positive contact with outgroup members significantly improves intergroup outcomes. Importantly, the present study, together with prior research in extreme intergroup contexts (e.g., Hewstone et al. 2014; Voci et al. 2015), shows that the benefits of positive contact are not confined to benign intergroup situations; rather, contact can foster improved outcomes even in more severe conflicts. Observations from historically divided regions such as Northern Ireland and South Africa further support this claim, showing that increased intergroup contact can gradually diffuse even deeply entrenched hostilities (Pettigrew et al. 2011). Notably, the present study focused on particularly demanding intergroup outcomes—trust and forgiveness—yet found medium contact effects. This, however, does not mean that positive intergroup contact is a panacea that can solve all conflicts. In fact, to avoid potential boomerang effects of contact (see Techakesari et al. 2015), it is advisable to first remove objective barriers (structural or institutional) to reconciliation, thereby creating the conditions for constructive contact to emerge.

Concerning the effect of intergroup apologies, previous experimental studies revealed mixed findings regarding apologies’ effects on forgiveness and trust. The present study suggests apologies’ effects are conditional upon several factors, including perceived sincerity and typicality, which can amplify or attenuate their impact, depending on the perceptions they generate. Regarding forgiveness, apologies significantly increased forgiveness toward the apologizing group when perceived as sincere (*d* = 0.52) (see also Wohl et al. 2013) and decreased it when perceived as insincere (*d* = –0.36), resulting in an almost null average effect (*d* = 0.08). A similar pattern has been documented in the interpersonal domain. For example, Darby and Schlenker (1989) found that when the apologizer was perceived as untrustworthy, offering an apology actually led to more negative evaluations than offering no apology at all. Similar conditional effects have been observed at the intergroup level. For example, Nadler and Liviatan (2006) found that expressions of empathy by an outgroup representative reduced willingness for reconciliation among participants with low trust in the outgroup, while Leonard et al. (2011) showed that the absence of an apology following a transgression increased victims’ desire for retribution and decreased forgiveness by eliciting emotions such as anger and diminished respect. Taken together, these findings may suggest that both failing to offer an apology when one is warranted and offering an apology that is perceived as insincere may evoke similar negative emotional reactions, thereby producing comparable adverse effects in intergroup contexts.

For trust, there was an overall significant effect of apology (*d* = 0.40) (see also Katz et al. 2008; Reinders Folmer et al. 2021), which was also influenced by both sincerity and typicality. The effect was strongest when apologies were seen as sincere (*d* = 0.82) and the representatives as typical (*d* = 0.71) but nearly null when perceived as insincere (*d* = –0.09) or non-typical (*d* = –0.01). These findings highlight the central role of victims’ perceptions and emphasize that an effective apology involves more than simply expressing regret for past transgressions.

It is important that apologies are offered based on moral grounds rather than on strategical motivations such as obtaining unilateral benefits. Apologies can raise victims’ expectations of genuine behavioral change (Wohl et al. 2011), yet this can only happen if the majority of the perpetrator’s group democratically supports the apology. An apology should therefore be offered only when it has the support of the majority of the perpetrator group and is delivered in a manner that reflects the group’s collective perspective. Apologies that are perceived as insincere or non-representative of the group’s views seem to be treated with a high degree of cynicism by the victim group and can even harm intergroup relations, as shown in the present study.

The present study did not find a significant interaction between intergroup apologies and intergroup contact, suggesting instead that the two factors might operate independently when it comes to the relationship between Romas and Romanians. A second explanation might lie in the low power of the study to detect interaction effects. This is most evident in the non-significant interaction between contact and apology in predicting forgiveness. Although the interaction coefficient was in the expected direction and of small-to-moderate magnitude (*β* = 0.21), it did not reach statistical significance. Future studies could reexamine this issue using larger samples to ensure sufficient statistical power to detect even small interaction effects.

One of the things that stand out in the literature is that, so far, research has focused exclusively on the outcomes of apologies coming from the perpetrator to the victim. Nevertheless, in many intergroup conflicts and especially in historical ones, these roles are more fluid. A group may have a history of victimization, yet it can also bear the responsibility for harm in specific episodes. This duality of roles has important implications for both the motivations underlying the decision to apologize and the expectations surrounding apologies from the perspective of the victimized group. The present study is, to my knowledge, the first one to test the effects of apologies offered by the less powerful group. It has been previously suggested that such apologies might be easily dismissed, as they fail to address the emotional needs of the more powerful group (Shnabel & Nadler 2008). Specifically, when a disadvantaged group apologizes, this act may further threaten its already fragile sense of power while simultaneously challenging the powerful group’s moral self-image, particularly if the latter has not previously apologized or acknowledged past wrongdoings. Nevertheless, the present findings indicate that apologies from less powerful groups can exert a meaningful positive effect, provided they are interpreted as authentic and representative rather than strategic or isolated gestures.

As Páez (2010) notes, apologies validate victims’ demands by acknowledging suffering that was previously ignored. Therefore, when both parties of the conflict have caused harm, they may both need to redeem themselves before intergroup relations can normalize and reconciliation can begin. More importantly, these findings indicate that in intergroup conflicts, less powerful groups do not need to wait for privileged groups to act but can play an active role and even take the initiative in the reconciliation process. However, these results do not imply that reconciliation necessitates or should prioritize apologies from structurally disadvantaged groups. The observation that less powerful groups can contribute to reconciliation processes must not be taken as a justification for inaction on the part of more powerful groups, nor should it legitimize expectations that marginalized groups assume responsibility for repairing intergroup relations. In contexts marked by structural inequality, such expectations risk reinforcing existing power asymmetries by shifting the moral burden onto those who are already disadvantaged while allowing dominant groups to evade responsibility for past or ongoing harm. Importantly, because such an apology may carry significant moral and historical implications for the less powerful group as well, it should ideally be discussed within the group and receive broad community support before being issued, in order to minimize potential backlash from members who may oppose their leaders’ decision to apologize. Accordingly, apologies from marginalized groups should be understood as meaningful only when they are voluntary, democratically supported, free from external pressure, and not accompanied by implicit or explicit moral responsibility. Finally, reconciliation efforts must remain attentive to structural injustices and ensure that gestures of goodwill do not mistakenly reproduce the very inequalities they seek to address.

### 4.1. Limitations and recommendations for future studies

The present study has some important limitations that are worth mentioning. First, it might suffer from methodological limitations. Specifically, intergroup contact, sincerity, and typicality were measured rather than experimentally manipulated, meaning that conclusions about their impact on the studied outcomes are subject to the usual constraints of correlational research. In addition, sincerity and typicality were measured with only a single item, which may limit the reliability of their operationalization, while intergroup contact was self-reported. Consequently, participants may have been biased in their estimates of how much and how positive their contact with Romas was in the past. Nevertheless, such bias is likely limited, as evidence shows that self-reported measures are still valid measures of contact (see Hewstone et al. 2011). Furthermore, participants were assumed to possess a collectivistic orientation, although their degree of collectivism was not measured. Future studies could incorporate such measures to provide stronger support for the interpretations offered.

Second, the manipulation may suffer from ecological validity limitations. A written article does not necessarily carry the same impact as an apology delivered through official channels by high-status representatives. Additionally, broader social dynamics unfolding after an apology, such as media coverage or public reactions, may amplify or attenuate its effects. Future research could examine the potential influence of contextual realism on recipients’ reactions following an apology. The timing of measurement may also influence some conclusions, as we assessed the dependent variables immediately after the manipulation. Several authors (e.g., McCullough et al. 2003; Philpot & Hornsey 2008; Sells & Hargrave 1998) have argued that apologies may exert delayed rather than immediate effects on intergroup outcomes, potentially producing stronger impacts over time. However, the opposite may also occur; within the context of an ongoing intergroup conflict, the effect of an apology may fade quickly or be easily forgotten (see Philpot & Hornsey 2011). Future longitudinal research should therefore explore how the impact of intergroup apologies evolves and identify the mediating and moderating factors that shape these temporal dynamics.

Third, it is important to acknowledge that the control condition consisted of a simple no-apology text, which does not entirely rule out alternative explanations for the observed effects, such as demand characteristics or priming effects. Finally, as these findings were obtained in a specific socio-cultural context and based on a non-representative sample, they should be replicated in other intergroup conflict settings to determine whether the effects are robust and generalizable.

### 4.2. Conclusion

The results of this study suggest that apologies from low-power groups can produce positive intergroup effects, thereby showing that such groups can play an active role in reconciliation. Such apologies seem to be most effective when they are perceived as sincere and when the individuals issuing them are viewed as representatives of their group. In practical terms, this implies that reconciliation efforts should pay close attention not only to the content of the message but also to how apologies are communicated and by whom. At the same time, given the sensitive nature of such gestures, it is recommended that they be offered voluntarily, be supported by the group, and remain free from external pressure in order to avoid opposition within the group or reinforcing existing power inequalities.

## Data Accessibility Statement

The dataset generated for the current study is publicly available in the OSF Repository at https://osf.io/9c5az/.

## Additional File

The additional file for this article can be found as follows:

10.5334/irsp.1157.s1 Supporting Information. Apology manipulation and items of the scales measuring trust and forgiveness.
