# Supplementary material for: When the Underdog Apologizes: The Role of Intergroup Apologies in Historical Intergroup Conflicts
Source: Int Rev Soc Psychol. 2026 Apr 8;39:3. doi: 10.5334/irsp.1157 (PMC13068085; doi:10.5334/irsp.1157)
Supplement: Supporting Information. — Apology manipulation and items of the scales measuring trust and forgiveness. [file irsp-39-1157-s1.pdf]

Supporting Information

The text of the article that was used as manipulation, translated from Romanian:

A historical moment: Romas apologized!

On 10th April, at the Congress for Human Rights held at Intercontinental Hotel in Bucharest, an unimaginable event for many Romanians happened, which could mark a turn in the relations between Romanians and Romas: Romas' Federation in Romania has issued and signed a collective statement apologizing in the name of all Romas for the damages caused to Romanian people. As the spokesperson Aurel Trandafir himself declared: "We, the Romas, apologize to all Romanians and to the Romanian society at large for the damages that Roma ethnics have caused to Romanians. We are truly sorry for the negative image that Romanians have acquired in Western Europe, which is partly influenced by the behavior of certain Roma ethnics abroad, a behavior that we Romas strongly condemn".

Their surprising statement continued, in the amazement of the people present at the event: "Romas are a strong and proud people and saying sorry is not something that we do with ease. Nevertheless, we would like to express our remorse today for the harm and injustices suffered by many Romanians, which were caused by Roma ethnics. We are sad that due to the immoral or illegal behavior of some Romas a lot of honest Romanians have had to suffer emotional or economical costs and that these types of behaviors have also deteriorated the relations between our two ethnicities."

At the end of their statement, Romas have expressed through Mr. Transafir's voice their appreciation for Romanian culture and their hope for more harmonious relations with Romanians: "Romas have a deep respect for Romania, Romanians and the rich Romanian culture. We believe that both Romas and Romanians can learn from each other and that a strong

and positive relationship between our two ethnicities is entirely possible. Therefore, we truly hope that our gesture today is a first step in forming such a relationship in the future."

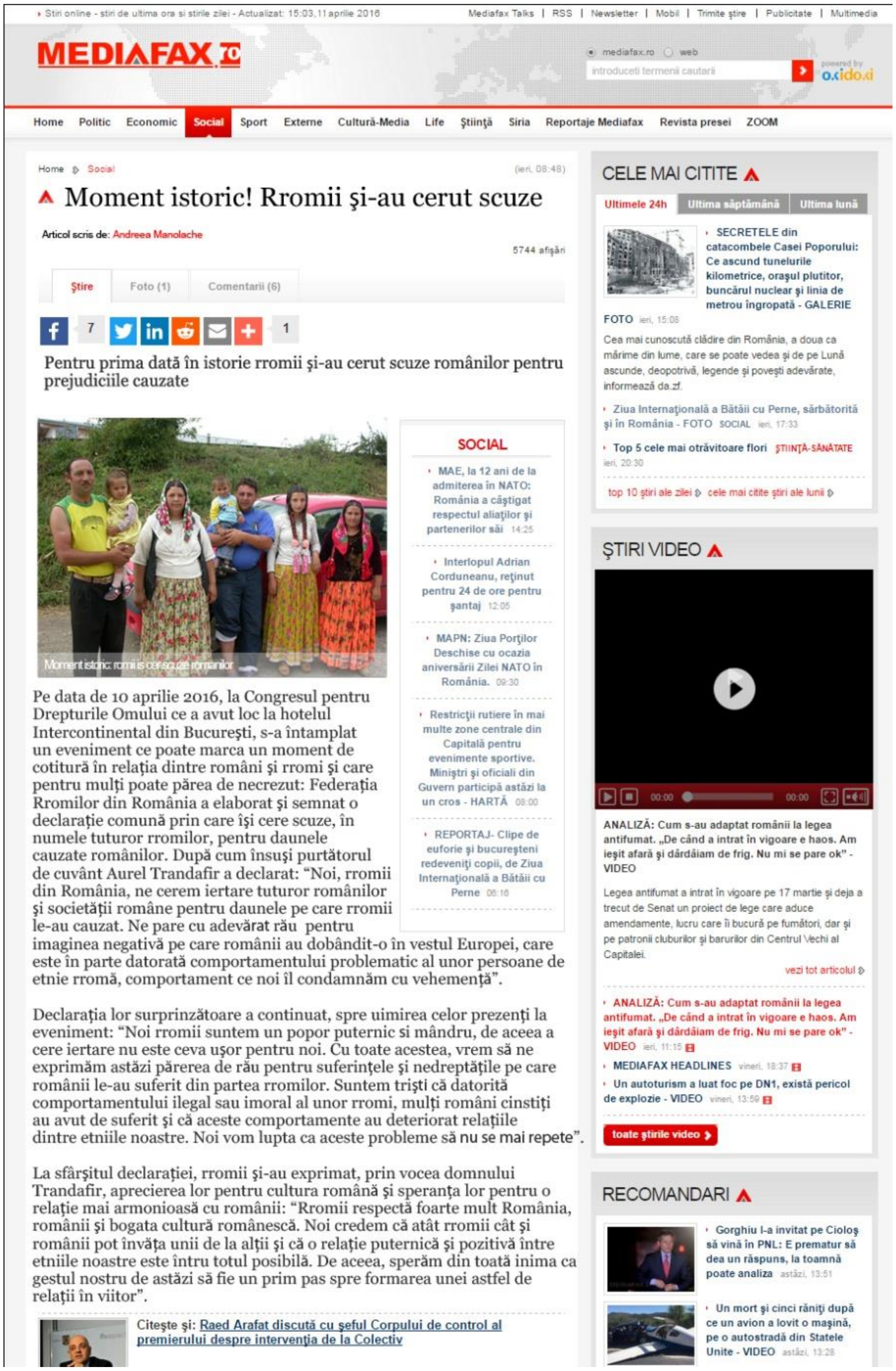

Stiri online - stiri de ultima ora si stirile zilei - Actualizat: 15:03,11 aprilie 2016 | Mediafax Talks | RSS | Newsletter | Mobil | Trimite știre | Publicitate | Multimedia

MEDIAFAX.ro

Home | Politic | Economic | Social | Sport | Externe | Cultură-Media | Life | Știință | Siria | Reportaje Mediafax | Revista presei | ZOOM

Home > Social (ieri, 08:48)

Moment istoric! Rromii şi-au cerut scuze

Articol scris de: Andreea Manolache

5744 afişări

Ştire | Foto (1) | Comentarii (6)

Pentru prima dată în istorie rromii şi-au cerut scuze românilor pentru prejudiciile cauzate

Moment istoric: rromii is cer scuze romanilor

Pe data de 10 aprilie 2016, la Congresul pentru Drepturile Omului ce a avut loc la hotelul Intercontinental din Bucureşti, s-a întamplat un eveniment ce poate marca un moment de cotitură în relația dintre români şi rromi şi care pentru mulți poate părea de necrezut: Federația Rromilor din România a elaborat şi semnat o declarație comună prin care îşi cere scuze, în numele tuturor rromilor, pentru daunele cauzate românilor. După cum însuşi purtătorul de cuvânt Aurel Trandafir a declarat: "Noi, rromii din România, ne cerem iertare tuturor românilor şi societății române pentru daunele pe care rromii le-au cauzat. Ne pare cu adevărat rău pentru imaginea negativă pe care românii au dobândit-o în vestul Europei, care este în parte datorată comportamentului problematic al unor persoane de etnie rromă, comportament ce noi îl condamnăm cu vehemență".

Declarația lor surprinzătoare a continuat, spre uimirea celor prezenți la eveniment: "Noi rromii suntem un popor puternic si mândru, de aceea a cere iertare nu este ceva uşor pentru noi. Cu toate acestea, vrem să ne exprimăm astăzi părerea de rău pentru suferințele şi nedreptățile pe care românii le-au suferit din partea rromilor. Suntem trişti că datorită comportamentului ilegal sau imoral al unor rromi, mulți români cinstiți au avut de suferit şi că aceste comportamente au deteriorat relațiile dintre etniile noastre. Noi vom lupta ca aceste probleme să nu se mai repete".

La sfârşitul declarației, rromii şi-au exprimat, prin vocea domnului Trandafir, aprecierea lor pentru cultura română şi speranța lor pentru o relație mai armonioasă cu românii: "Rromii respectă foarte mult România, românii şi bogata cultură românescă. Noi credem că atât rromii cât şi românii pot învăța unii de la alții şi că o relație puternică şi pozitivă între etniile noastre este întru totul posibilă. De aceea, sperăm din toată inima ca gestul nostru de astăzi să fie un prim pas spre formarea unei astfel de relații în viitor".

Citeşte şi: Raed Arafat discută cu şeful Corpului de control al premierului despre intervenția de la Colectiv

SOCIAL

- MAE, la 12 ani de la admiterea în NATO: România a câştigat respectul aliaților şi partenerilor săi 14:25
- Interlopul Adrian Corduneanu, reținut pentru 24 de ore pentru şantaj 12:05
- MAPN: Ziua Porților Deschise cu ocazia aniversării Zilei NATO în România. 09:30
- Restricții rutiere în mai multe zone centrale din Capitală pentru evenimente sportive. Miniştri şi oficiali din Guvern participă astăzi la un cros - HARTĂ 08:00
- REPORTAJ- Clipe de euforie şi bucureşteni redeveniți copii, de Ziua Internațională a Bătăii cu Perne 06:16

CELE MAI CITITE

Ultimele 24h | Ultima săptămână | Ultima lună

- SECRETELE din catacombele Casei Poporului: Ce ascund tunelurile kilometrice, oraşul plutitor, buncărul nuclear şi linia de metrou îngropată - GALERIE FOTO ieri, 15:08

Cea mai cunoscută clădire din România, a doua ca mărime din lume, care se poate vedea şi de pe Lună ascunde, deopotrivă, legende şi poveşti adevărate, informează da.zf.

- Ziua Internațională a Bătăii cu Perne, sărbătorită şi în România - FOTO SOCIAL ieri, 17:33
- Top 5 cele mai otrăvitoare flori ŞTIINŢĂ-SĂNĂTATE ieri, 20:30

top 10 ştiri ale zilei | cele mai citite ştiri ale lunii

ŞTIRI VIDEO

ANALIZĂ: Cum s-au adaptat românii la legea antifumat. „De când a intrat în vigoare e haos. Am ieşit afară şi dârdâiam de frig. Nu mi se pare ok" - VIDEO

Legea antifumat a intrat în vigoare pe 17 martie şi deja a trecut de Senat un proiect de lege care aduce amendamente, lucru care îi bucură pe fumători, dar şi pe patronii cluburilor şi barurilor din Centrul Vechi al Capitalei.

vezi tot articolul

- ANALIZĂ: Cum s-au adaptat românii la legea antifumat. „De când a intrat în vigoare e haos. Am ieşit afară şi dârdâiam de frig. Nu mi se pare ok" - VIDEO ieri, 11:15
- MEDIAFAX HEADLINES vineri, 18:37
- Un autoturism a luat foc pe DN1, există pericol de explozie - VIDEO vineri, 13:59

toate ştirile video

RECOMANDARI

- Gorghiu l-a invitat pe Cioloş să vină în PNL: E prematur să dea un răspuns, la toamnă poate analiza astăzi, 13:51
- Un mort şi cinci răniți după ce un avion a lovit o maşină, pe o autostradă din Statele Unite - VIDEO astăzi, 13:28

Image 1: The apology manipulation as it was received by the participants.

Table 1: Items of the scales for forgiveness and trust.

Forgiveness	
	It is important that Romanians never forgive the wrongs done by the Romas (R)
	Personally, I forgive Romas for the harm they caused to Romanians
	It is not possible for me to forgive Romas for the harm they caused to Romanians (R)
Trust	
	Most members of the Roma community try to be fair
	Few Romas can be trusted (R)
	Most members of the Roma community cannot be trusted to deliver on their promises (R)
